# O03 ST97 *Staphylococcus aureus* and oxacillin resistance: an emerging challenge for microbiologists?

**DOI:** 10.1093/jacamr/dlac052.003

**Published:** 2022-05-31

**Authors:** G Hughes, D Li, R Kerrigan, I Gupta, S Wilkinson

**Affiliations:** UKHSA lab, Birmingham, B9 5SS, UK

## Abstract

**Background:**

The presence of cefoxitin and oxacillin resistance in *Staphylococcus aureus* isolates is suggestive of methicillin resistance (MRSA) with molecular detection of the *mecA* or *mecC* genes providing confirmation. The UK SMI and EUCAST guidelines for MRSA detection describe a subset of strains that exhibit reduced susceptibility to oxacillin and cefoxitin, though negative for *mecA* and *mecC* genes—frequently named borderline oxacillin-resistant *S. aureus* (BORSA). Local data noted a number of *S. aureus* isolates that matched the above phenotype leading to laboratory, clinical and epidemiological questions.

**Methods:**

Routine samples were collected between August 2020 and April 2021 at West Midlands Health Security Agency Laboratory, Birmingham. If provisional results suggested an MRSA isolate, the following confirmatory tests were undertaken. Locally, clinical samples underwent susceptibility testing with cefoxitin disc diffusion and oxacillin gradient diffusion (MRSA screens) or Vitek 2 (non-MRSA screens). Isolates with discrepant results [susceptible to cefoxitin; resistant to oxacillin (MIC >2 mg/L) or vice versa] were sent to the reference laboratory at Colindale, UK for further testing, which included *mecA* or *mecC* gene detection by PCR and serotyping with Illumina sequencing for all isolates.

**Results:**

In total, 53 isolates were sent to the reference lab of which 22 were confirmed to have an ST97 serotype. All 53 were negative by PCR for *mecA* and *mecC* genes. For the 22 isolates of ST97, local oxacillin MIC values ranged from 2 to 6 mg/L. Cefoxitin susceptibility was confirmed locally in all isolates. The 22 isolates were from 15 patients; wound swabs (*n* = 18); blood culture (*n* = 1), sputum (*n* = 1) and MRSA screen (*n* = 2). Median patient age was 48 years (IQR 38–59) with 5 being female and 10 male. A history of injecting drug use was documented in 53% (8/15).

**Conclusions:**

BORSA remains a problem from a laboratory, clinical and infection control perspective. This work raises two important questions: what is the most appropriate local laboratory testing pathway and what is the clinical relevance of these isolates (i.e. can flucloxacillin be relied upon in treatment)? The ST97 serotype appears to be associated with skin and soft tissue infection and may be linked to people who inject drugs.

